# Cost-Effectiveness in Alternative Treatment Options for Pancreatic Pseudocysts

**DOI:** 10.3390/reports7020038

**Published:** 2024-05-17

**Authors:** Nikola Boyanov, Nikol Milinich, Katina Shtereva, Katerina Madzharova, Stoilka Tufkova, Mariana Penkova-Radicheva, Daniela Radicheva, Neno Shopov

**Affiliations:** 1 Medical Simulation Training Center, Research Institute, Medical University of Plovdiv, 4000 Plovdiv, Bulgaria; nikolaboyanov@gmail.com (N.B.); tuffi.med@gmail.com (S.T.); 2 Department of Gastroenterology, Pulmed University Hospital, 4000 Plovdiv, Bulgaria; katinashtereva@gmail.com (K.S.); katerina.madzarova@gmail.com (K.M.); 3 Department of Clinical Toxicology, St. George University Hospital, 4000 Plovdiv, Bulgaria; 4 Second Department of Internal Medicine Therapy, Medical Faculty, Trakia University, 6000 Stara Zagora, Bulgaria; mpenkovadoc@abv.bg (M.P.-R.); d_radicheva@abv.bg (D.R.); 5 Department of Surgery, Pulmed University Hospital, 4000 Plovdiv, Bulgaria; neno_shopov@yahoo.com

**Keywords:** cold LAMS, endoscopic ultrasound-guided drainage, EUS-guided drainage, EUS, hot LAMS, low cost, lumen-apposing metal stent, nasocystic catheter, pancreatic pseudocyst, pancreatitis, transgastric drainage

## Abstract

**Background and Objectives**: Pancreatic pseudocysts often arise as complications of pancreatitis and present unique challenges in clinical management, encompassing considerations for both technical aspects and financial implications. Before the advancements of invasive gastroenterology, pancreatic pseudocysts have been drained surgically for many years. Nowadays, we have less invasive techniques with higher efficiency and lower mortality rates, however, they remain cost-challenging for most countries. **Materials and Methods**: We present four patients (two males and two females) with pancreatic pseudocysts who underwent endoscopic ultrasound-guided transgastric drainage using plastic stents accompanied by a standard lavage protocol using a nasocystic catheter. **Results**: All four patients had successful outcomes, and a follow-up at 6 months revealed no traces of the pseudocysts or any significant long-term complications. One acute complication (arterial bleeding) and one late complication (stent migration) were observed. As the study aimed to present a cheaper option for draining pancreatic pseudocysts, we investigated and compared costs for the materials we utilized and those associated with lumen-apposing metal stents. Upon compiling the data, a notable advantage was evident in favour of our method. **Conclusions**: While EUS-guided drainage of pancreatic pseudocysts using lumen-apposing metal stents (LAMSs) represents a high-end strategy for treating pancreatic pseudocysts, our method demonstrates better cost-effectiveness without compromising efficacy.

## 1. Introduction

Pancreatic pseudocysts (PPs) are encapsulated fluid collections, with little or no presence of necrotic tissue, that arise from the pancreas as a complication of acute or chronic pancreatitis, more often the latter one. The term “pseudo” refers to the non-epithelialized wall made up of fibrous and granulation tissue that surrounds the fluid collection. The main causative factors include alcohol abuse, biliary stones, high triglycerides, trauma, post-ERCP/surgery, and idiopathic origins [1].

The choice of therapeutic approach is wholly contingent upon both the underlying cause and the unique profile of the individual patient. Pseudocysts that form after acute pancreatitis normally undergo spontaneous resolution without intervention within 4 to 6 weeks. In contrast, those associated with chronic pancreatitis, characterized by a mature cyst wall, seldom resolve on their own.

Indications for invasive drainage of pancreatic pseudocysts are persistent pain unresponsive to medications, obstruction causing issues with gastric or duodenal passage, obstruction of the common bile duct due to external compression, obstruction of the excretory system, infected pancreatic pseudocysts, compression of large vessels, intracystic hemorrhaging, pseudocyst rupture extending into neighboring organs or the abdominal cavity, fistualization of the pseudocyst to regions such as the pleura, mediastinum, or scrotum and pseudocysts > 6 cm or unchanged in size for more than 6 weeks or suspected malignancy [2].

Invasive treatments for pancreatic pseudocysts aim at draining or removing the fluid-filled sacs. They include percutaneous catheter drainage, surgical drainage, laparoscopic drainage, endoscopic transluminal drainage, ERCP transpapillary drainage, and endoscopic ultrasound-guided drainage (EUS-G drainage) [2,3,4]. Endoscopic ultrasound-guided drainage using metal or plastic stents has emerged as the preferred method due to its minimally invasive nature and notable long-term success rates. Lumen-apposing metal stents (LAMSs) are preferred over plastic stents due to their higher clinical success rate and lower complication rates [4]. However, their high cost can pose a challenge, particularly in countries where patients are responsible for covering the expenses of the materials used. The aim of this article is to demonstrate a cost-effective alternative treatment for pancreatic pseudocysts that does not involve the use of LAMSs.

## 2. Detailed Case Description

### 2.1. Materials and Methods

Inclusion criteria were accessibility to the pseudocyst, platelet count of >50 × 10^9^/L, wall thickness between >3 mm and <1 cm, distance of the pseudocyst to the gastrointestinal wall < 1 cm, minimum size of the pseudocyst 5–6 cm, and last but not least, informed consent from the participants [5].

Exclusion criteria were contraindications for EUS (esophageal and duodenal stenosis, suspected perforation, ingested corrosive agent during the first 24 h, ileus, severe heart failure, severe respiratory failure, active tuberculosis, and dissecting aortic aneurysm), difficulty in the visualization of the target, coagulation abnormalities, severe acute pancreatitis, sepsis, unstable cardiovascular status, and patient’s refusal or inability to cooperate.

Written consent was obtained from all patients before the procedure. Each patient was admitted one day before the procedure was carried out, to ensure stable vital signs and lab results. A sensitivity test for Urografin was performed on each of the patients prior to the procedure and antibiotic therapy with meropenem was initiated preemptively to minimize the risk of infection.

The EUS-guided drainage was performed using the Hitachi Arietta 850 as the ultrasound device and Pentax Medical EG38-J10UT ultrasound video gastroscope. All procedures were carried out under general anesthesia with propofol, fentanyl, midazolam, atracurium, suxamethonium, and sevoflurane.

**Figure 1:**
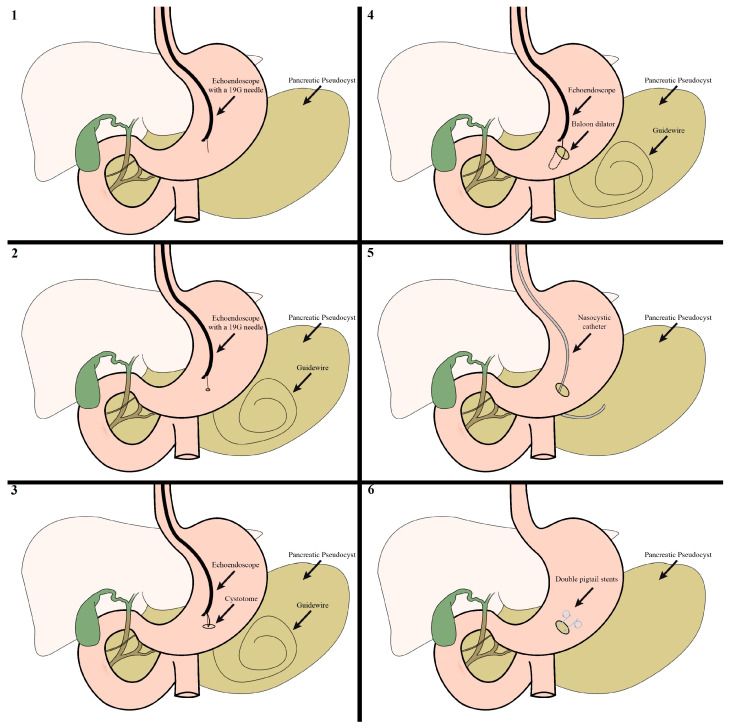
The step-by-step process of echoendoscopic drainage of pancreatic pseudocysts: 1—puncturing the pseudocyst with a 19-G needle; 2—inserting a guidewire until it was coiled into at least two loops; 3—widening the tract with a cystotome; 4—dilating the tract with a balloon dilator; 5—nasocystic catheter used for irrigation of the pseudocyst; 6—two double pigtail stents.

The drainage was conducted using CO_2_ insufflation to reduce the risk of complications and was guided by both fluoroscopy and echoendoscopy. The procedural steps are shown in Figure 1. The optimal location for draining the pancreatic pseudocyst was determined using the echoendoscope while simultaneously ensuring the absence of blood vessels in the vicinity using Doppler imaging. A 19G needle was then advanced through the wall of the stomach into the pseudocyst.

**Figure 2:**
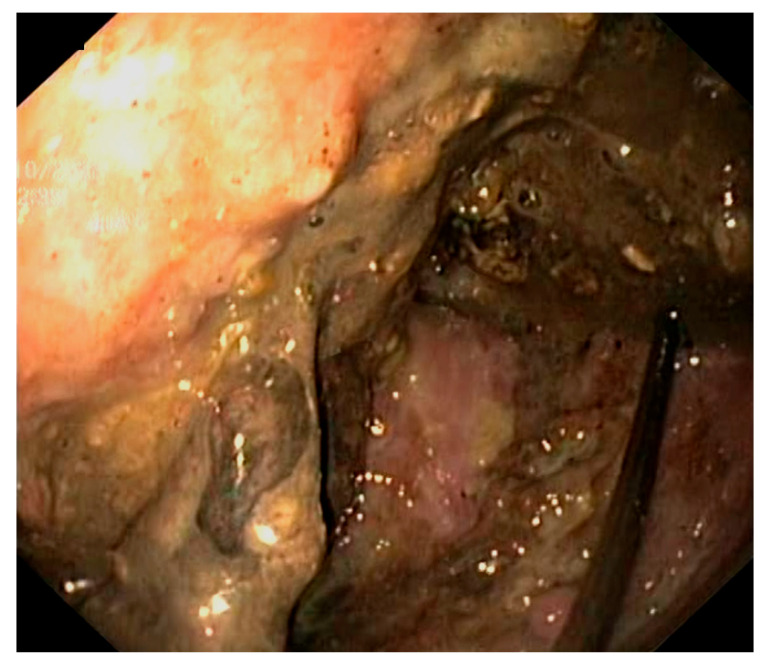
Pancreatic pseudocyst with necrotic tissue.

Fluid from the pseudocyst was aspirated through the needle and sent for analysis, including microbiology, cytology and biochemistry. Afterward, an Urografin solution was sprayed through the needle, followed by an X-ray examination to verify the positioning of the needle inside the pseudocyst. Under fluoroscopic guidance, a guidewire was then passed through the needle and into the pseudocyst until it was coiled into at least two loops, ensuring a pathway for the subsequent steps. The needle was then removed. Over the guidewire, the entry tract was widened using a 6 Fr cystotome and then dilated with a balloon dilator up to 12 mm in three of the cases and 15 mm in the fourth one. At this point, a sudden discharge of pus was observed in 3 of the cases. Afterward, the endoscope was used to check the dilated zone in case there was bleeding and then inserted into the pseudocyst to assess the presence of necrotic tissues. Each of the four cases exhibited a significant amount of necrosis, which is why we decided to put in a nasocystic drainage catheter before performing necrosectomies (Figure 2).

**Figure 3:**
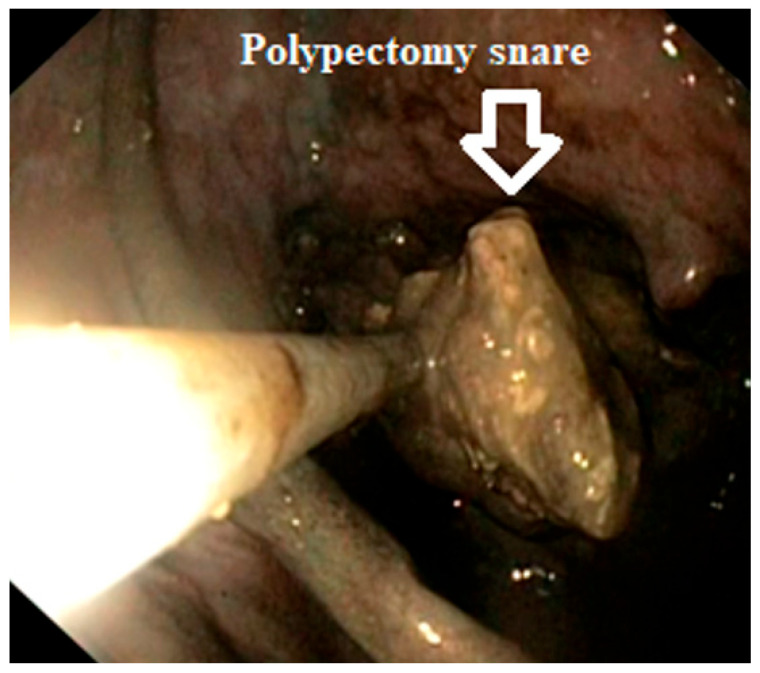
Necrosectomy using a polypectomy snare inside of a pancreatic pseudocyst.

Under fluoroscopic guidance, a thin nasocystic catheter was inserted into the pseudocyst using the guidewire. With the help of a nasogastric tube, the position of the drainage was converted from the mouth to the nose. Initially, an irrigation procedure was performed using a mixture of 150 mL of hydrogen peroxide (H_2_O_2_) and saline solution (0.9% NaCl) in a 1:2 ratio, and then an additional lavage was conducted using 750 mL of saline alone. Over the next 2 to 3 weeks, a lavage of 200 mL was administered 3 to 4 times a day, using a mixture of hydrogen peroxide (H_2_O_2_) to a normal saline solution at a 1:3 ratio. Once the patients experienced improvement in both their clinical condition and laboratory parameters, a follow-up EUS was conducted to assess the pseudocyst. All four patients still had necrotic tissues present and therefore each of them underwent 1 to 4 necrosectomies using a polypectomy snare (Figure 3).

Only after ensuring all the necrotic tissue had been removed, the nasocystic drainage was extracted. Two 7 Fr/4 cm double pigtail stents were then introduced over a guidewire under fluoroscopic guidance in order to maintain a continuous drainage pathway from the pseudocyst to the gastrointestinal tract.

There was no clinical data for a medical risk at the time of discharge for any of the patients.

### 2.2. Results

**Figure 4:**
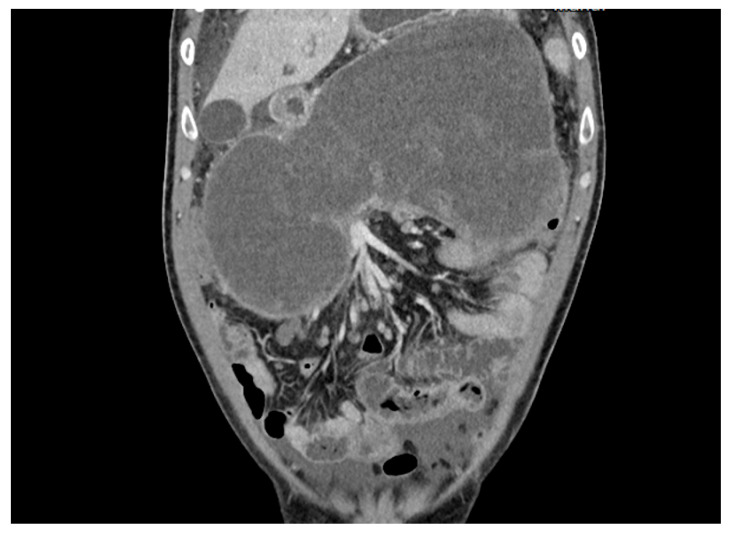
CT scan of a 35-year-old patient with a pancreatic pseudocyst.

Between August 2023 and October 2023, in Pulmed University Hospital in Plovdiv, Bulgaria, four patients presented with pancreatic pseudocysts (Figure 4), two of whom were males, and two were females (Table 1). The age range varied between 34 and 82 years old.

All four patients developed pancreatic pseudocysts after acute pancreatitis: one due to chronic alcohol abuse and the remaining three due to choledocholitiasis. Two patients presented with pancreatic pseudocysts located in the body and tail of the pancreas, whereas the remaining two patients had pseudocysts distributed across the head, body, and tail of the pancreas.

Among the four cases of pancreatic pseudocysts, several symptoms were commonly observed (Table 2). Abdominal pain emerged as the most prevalent symptom, occurring in all cases. It was accompanied by back pain and malaise, noted in three out of the four cases. Palpable abdominal masses were detected in only two of the cases, due to the lower BMI observed in comparison to the other two patients.

**Figure 5:**
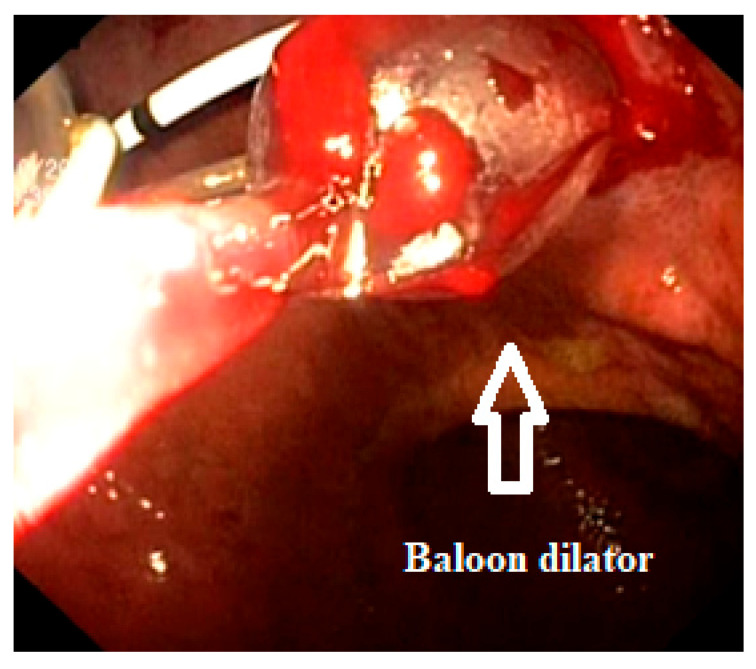
Active bleeding during the dilation of the gastrocystic opening.

A success rate of 100% was achieved in all of the cases, with one acute and one delayed complication observed. Arterial bleeding occurred during the dilation of the gastrocystic opening in one of the cases. Despite an initial attempt to stop the bleeding using a coagulation grasper, it persisted, prompting the placement of a 16 mm Lockado hemostatic clip (Figure 5).

**Figure 6:**
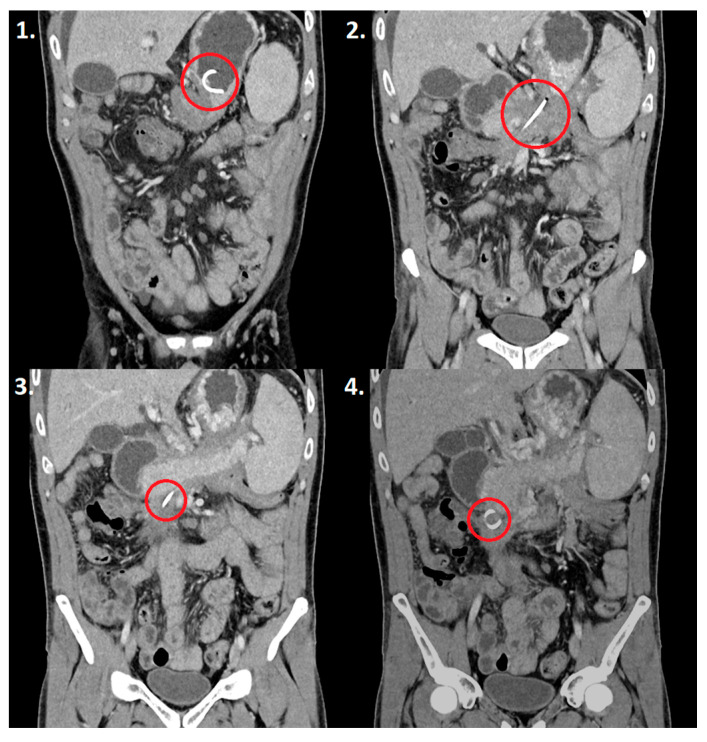
CT scan showing a single double-pigtail stent during one of the patient follow-ups 6 months after the procedure (in the red circle images 1–4).

Follow-up assessments were performed on each patient six months later and none of them exhibited a recurrence of pancreatic pseudocysts or any symptoms during the recovery period. In only one of the four patients a late complication was observed—stent migration outside of the cyst. To exclude the possibility of the stent being lodged within the pseudocyst, we performed an X-ray examination. The presence of the second stent ensured that the gastrocystostomy remained open, thereby averting any adverse consequences (Figure 6).

The mean hospital stay was 17 days (range, 9–28 days), and none of the patients required monitoring in an ICU unit or an emergency surgery.

We calculated the approximate material costs, when using EUS-guided transgastric drainage with a nasocystic catheter (Table 3) and when using hot and cold LAMS (Table 4). In the first case, the price solely for the materials amounts to approximately EUR 1200, whereas the other two cost around EUR 4500 and EUR 2000.

## 3. Discussion

The likelihood of complications arising from EUS drainage depends on various elements, such as the proficiency of the healthcare professionals involved, the particular methodology employed, and the traits specific to each patient. Procedure-related complications range from 5% to 30%. Some of the most common complications are bleeding, infection, perforation, pancreatitis, leakage, stent-related complications and recurrence of the pseudocyst [4].

Stent migration is one of the most prevalent complications. It includes the migration of the stent inside or outside of the pancreatic pseudocyst, the blockage of the stent, etc. It is crucial to differentiate between early stent migration, which necessitates reintervention due to the disrupted drainage of the PP, whereas late stent migration is just a finding and does not require further action [5,6]. We opted for the application of two double- pigtail stents as a precautionary measure aimed at mitigating the potential risk of stent migration. Upon conducting follow-up assessments 6 months later one of the patients had a single double pigtail stent migrated, however, the drainage of the pseudocyst was unaffected as the second stent remained in place.

Bleeding also stands out as one of the most frequent complications and is observed in 1–10% of the patients, with severe bleeding in less than 1% [4,5]. It can occur at the needle puncture site, during the balloon dilation or during necrosectomies. Even with the utilization of the color Doppler, there is always the possibility of puncturing a small vessel, especially in large pseudocysts, because they may lead to portal vein hypertension and transmural varicose veins [6].

When it comes to discussing the utilization of EUS-guided transgastric drainage with plastic stents and a nasocystic catheter compared to lumen-apposing metal stents for treating pancreatic pseudocysts, it is essential to consider various factors like cost efficiency, efficacy and safety. They both emerged as high-end minimally invasive techniques over the last few years, however, in middle- and low-income countries, the economic aspect of these procedures is a priority. While metal stents might lead to fewer complications due to their larger luminal diameter and bi-flared flanges, these advantages are not prevalent enough in routine drainage procedures to justify the investment [7]. As shown in Table 3 and Table 4, the usage of cold LAMSs is associated with a price increase of over 60%, while the cost of using hot LAMSs increases by over 270% compared to plastic stents. We prefer the use of EUS-guided transgastric drainage with plastic stents and a nasocystic catheter as opposed to metal stents not only due to its cost-effectiveness but also because both methods achieve equivalent success rates. In a meta-analysis comparing the usage of double pigtail plastic stents to LAMSs, both groups demonstrated a technical success rate of 97.5% vs. 97.6% and no significant difference in the occurrence of complications [6,8,9]. The average duration of hospital stay remains relatively consistent across all approaches, further supporting the rationale for opting for the most cost-effective option [10]. This decision ensures that patients receive effective treatment without unnecessary financial burden. It is crucial to emphasize the significant role of the nasocystic drain in pseudocyst drainage when necrotic tissue is present. The drainage allows for the utilization of hydrogen peroxide to chemically debride the solid matter components within the pseudocyst, thus aiding in its resolution. In a retrospective study involving 87 patients, it was found that complete resolution using plastic stents alone was achieved in 58% of the cases, whereas the addition of nasocystic drainage increased the rate up to 79% [10].

When compared to surgical drainage, the EUS-guided drainage with a nasocystic catheter demonstrates several advantages. The most compelling one lies in its minimally invasive nature, which carries a lower risk of complications compared to traditional surgical interventions, shorter hospital stays and a faster recovery period following the hospital discharge. This not only reduces healthcare costs but also minimizes the risk of hospital-related complications such as infections and hospitalization-induced mental distress. Moreover, shorter hospital stays result in reduced medical leave durations, thereby offering economic benefits to the government. A systematic review of ten comparative studies shows that the success rate between surgical and EUS-guided approaches is similar, however, the latter has a reduced hospital stay, lower procedural costs, and improved quality of life [11]. Another systematic review involving 342 patients reveals reduced adverse effects in the endoscopic group, with a rate of 11.5% compared to 19.7% in the surgical group. Additionally, the former group demonstrates shorter hospital stays ranging from 0 to 25 days, whereas the surgical group’s stays span from 3 to 82 days [12].

## 4. Conclusions

In conclusion, managing pancreatic pseudocysts requires a multifaceted approach that considers the unique challenges and resource constraints prevalent in some countries. While advanced diagnostic and therapeutic modalities are integral, there is a pressing need for strategies that are cost-effective, sustainable, and accessible. The EUS-guided transgastric drainage with plastic stents and a nasocystic catheter emerges as the preferred option over hot and cold LAMSs providing significantly reduced procedural cost. While metal stents offer advantages such as larger luminal diameters and bi-flared flanges, the substantial cost increase associated with their usage outweighs these benefits in routine drainage procedures. This especially applies in countries where patients need to cover the costs of the materials used. Our preference for plastic stents and a nasocystic catheter is supported by equivalent success rates and comparable complication rates, as evidenced by recent meta-analyses [9]. Additionally, the role of the nasocystic drainage in the resolution of pseudocysts with necrotic tissue further underscores the versatility of this approach. By prioritizing cost-effectiveness and patient outcomes, clinicians can ensure that effective treatment is accessible to all individuals, without imposing unnecessary financial burden.

Lastly, EUS-G transgastric drainage with a nasocystic catheter offers several advantages over surgical drainage. Its minimally invasive nature reduces the risk of complications, leads to shorter hospital stays, faster recovery periods, offers economic benefits in terms of reduced medical leave durations and lower healthcare costs [11,12].

Overall, EUS-guided drainage with plastic stents and a nasocystic catheter represents a safe and cost-effective alternative to the treatment of pancreatic pseudocysts.

## Figures and Tables

**Table 1:** Patients’ data.

Case	Age	Gender	Hospital Stay (in Days)	Cause of PP	Location of PP	Size of PP (in mm)
1	34	Male	9	Biliary stones	Body, tail	154 × 66 mm
2	35	Male	28	Alcohol abuse	Head, body, tail	20 cm
3	60	Female	17	Biliary stones	Body, tail	111 × 70 mm
4	82	Female	17	Biliary stones	Head, body, tail	144 × 116 mm

**Table 2:** Patients’ symptoms.

Symptoms	Case 1	Case 2	Case 3	Case 4
Abdominal pain	Yes	Yes	Yes	Yes
Back pain	-	Yes	Yes	Yes
Palpable abdominal mass	Yes	Yes	-	
Early satiety	-	Yes	-	Yes
Nausea and vomiting	-	Yes	-	Yes
Loss of weight	-	Yes	-	Yes
Fever	Yes	-	Yes	-
Jaundice	-	Yes	-	-
Malaise	-	Yes	Yes	Yes

**Table 3:** Material costs for EUS-guided transgastric drainage with a nasocystic catheter.

Materials	Cost per 1 (EUR)	Number Used	Cost per Total Used (EUR)
19G Tru-cut needle	25.52	1	25.52
Guidewire	193.73	1	193.73
Cystotome	288.83	1	288.83
Balloon dilator	214.91	1	214.91
Nasocystic catheter	127.85	1	127.85
IV catheter	0.20	1	0.20
Nasogastric tube	0.32	1	0.32
Double-pigtail 7Fr/4 cm	54.22	2	108.44
Stent pusher catheter	81.17	2	162.34
Polypectomy snare	27.91	3	83.73
Total (EUR)			1205.87

**Table 4:** Material costs for EUS-guided transmural drainage by using hot and cold LAMS.

Materials	Cost per 1 (EUR)	Number Used	Cost per Total Used (EUR)
Hot LAMS	~4000	1	~4000
Cold LAMS	~1500	1	~1500
Stent pusher catheter	81.17	1	81.17
Nasogastric tube	0.32	1	0.32
Nasocystic catheter	127.85	1	127.85
IV catheter	0.20	1	0.20
Polypectomy snare	27.91	3	83.73
Double-pigtail 7Fr/4 cm	54.22	2	108.44
Foreign body retriever	38.64	1	38.64
Total (EUR) for hot LAMS			4440.35
Total (EUR) for cold LAMS			1940.35

## Data Availability

Data is contained within the article.
